# TRP Channels and Migraine: Recent Developments and New Therapeutic Opportunities

**DOI:** 10.3390/ph12020054

**Published:** 2019-04-09

**Authors:** Silvia Benemei, Greg Dussor

**Affiliations:** 1 Headache Centre, Careggi University Hospital, Viale Pieraccini 18, 50139 Florence, Italy; silvia.benemei@unifi.it; 2 School of Behavioral and Brain Sciences, Center for Advanced Pain Studies, The University of Texas at Dallas, Richardson, TX 75080, USA

**Keywords:** ion channel, TRP, cortical spreading depression, meninges, dura mater, botulinum toxin A, reactive oxygen species, reactive nitrogen species, neurogenic inflammation, CGRP

## Abstract

Migraine is the second-most disabling disease worldwide, and the second most common neurological disorder. Attacks can last many hours or days, and consist of multiple symptoms including headache, nausea, vomiting, hypersensitivity to stimuli such as light and sound, and in some cases, an aura is present. Mechanisms contributing to migraine are still poorly understood. However, transient receptor potential (TRP) channels have been repeatedly linked to the disorder, including TRPV1, TRPV4, TRPM8, and TRPA1, based on their activation by pathological stimuli related to attacks, or their modulation by drugs/natural products known to be efficacious for migraine. This review will provide a brief overview of migraine, including current therapeutics and the link to calcitonin gene-related peptide (CGRP), a neuropeptide strongly implicated in migraine pathophysiology. Discussion will then focus on recent developments in preclinical and clinical studies that implicate TRP channels in migraine pathophysiology or in the efficacy of therapeutics. Given the use of onabotulinum toxin A (BoNTA) to treat chronic migraine, and its poorly understood mechanism, this review will also cover possible contributions of TRP channels to BoNTA efficacy. Discussion will conclude with remaining questions that require future work to more fully evaluate TRP channels as novel therapeutic targets for migraine.

## 1. Migraine: Epidemiology and Symptoms

Migraine is one of the most common diseases worldwide affecting over 14% of the adult population [1], with a gendered distribution in favor of females (2:1), and an increased prevalence in reproductive age for both genders [2]. Importantly, migraine is the most common severe primary headache, characterized by long-lasting (4–72 hours) attacks of pain, accompanied by nausea, vomiting, and sensitivity to light and sound [1]. In current clinical practice, diagnosis of a migraine is made in accordance to the criteria of the International Headache Society (IHS) [3], which also allow the homogenous identification of the migraine phenotype worldwide for research purposes. According to the current version of the International Classification of Headache Disorders (ICHD-3) [3], the migraine can be with or without an aura (i.e., transient neurological symptoms, including visual or somatosensory or speech disturbances, that precede and/or accompany and/or follow the pain) and, in case of attacks for more than 15 days for 3 consecutive months, it can be classified as chronic. The most recent results from the Global Burden of Disease (GBD) study, promoted by the World Health Organization, reported migraine as the most disabling neurological disorder, and the second leading cause of years lived with disability (YLDs) worldwide [4]. The high disability, combined with the high prevalence, causes a significant socio-economic burden. In the European Union, where migraine is estimated to affect 75 million patients, each year it costs ~111 billion €, of which 93% is given for absence from work and reduced productivity [5].

## 2. Migraine: Therapeutics and Pathophysiology

Therapeutic options for the treatment of migraine include drugs for both the attacks, aimed to abort ongoing pain and reduce migraine-related symptoms, and prophylaxis. Acute treatment of attacks is recommended in any episode in any patient, while prophylaxis is recommended in the case of patients suffering more than four attacks/days of headache per month. Currently available and recommended drugs for the acute treatment of attacks encompass analgesics (i.e., paracetamol), non-steroidal anti-inflammatory drugs (NSAIDs), triptans, antiemetics and combinations of analgesics; while ergot derivatives and opioids maintain only a marginal role in therapy. Preventive treatments are to a large extent borrowed from other therapeutic areas, including antihypertensive agents (e.g., angiotensin-converting enzyme [ACE] inhibitors, angiotensin receptor blockers, beta blockers), antiepileptic drugs (e.g., topiramate, sodium valproate, gabapentin), tricyclic antidepressants (e.g., amitriptyline, nortriptyline), but there are also some drugs used only with migraine prevention objectives, such as flunarizine and pizotifene [1]. Importantly, recent clinical evidence has supported the inclusion of onabotulinum toxin A (BoNTA) among the recommended drugs for migraine prevention, but only for chronic patients [6,7].

Mechanisms contributing to migraine are multifactorial, complex, and not yet completely clarified [8,9,10]. Notwithstanding the huge number of studies, including genome-wide association studies (GWAS), the role of genetic factors still remains poorly defined [11], with the exception of hemiplegic migraine, which has, for a good portion of cases, a classical mendelian transmission. After years of open debate that has counter posed the neuronal and the vascular theory, migraine is now considered a neurovascular disorder, with a primary role of the trigeminovascular system in the mechanism of pain [12]. Following its first proposal, the neurovascular hypothesis has been corroborated by preclinical and clinical data showing the involvement of neurogenic inflammation and, in particular, of the neuropeptide calcitonin gene related peptide (CGRP) [12].

Neurogenic inflammation is a sterile inflammation mediated by the activation of trigeminal perivascular fibers that release neuropeptides, such as substance P and—most importantly in humans—CGRP, that induce increased blood flow and edema [12,13]. Importantly, it has been shown in preclinical models that the activation of meningeal nociceptors could stimulate the trigeminal ganglia [14], thus in turn perpetuating the release of vasoactive peptides, including CGRP.

## 3. CGRP and Migraine

CGRP, a neuropeptide produced from alternative splicing of the calcitonin gene, acts as a relevant signaling peptide in mammalian biology, with a crucial role in both physiological and pathological conditions. In particular, because of its potent action as a vasodilator, it is involved in the physiological regulation of vascular tone and blood pressure, and some evidence has been collected for a role in cardiovascular diseases and extra-cardiovascular conditions [15].

There are two CGRP forms differently expressed in humans, alpha-CGRP (primary sensory neurons of the dorsal root ganglia, vagal ganglia and trigeminal system) and beta-CGRP (intrinsic enteric neurons) [16,17,18]. According to the expression pattern, alpha-CGRP is primarily involved in migraine pathogenesis. CGRP acts via a G protein-coupled receptor constituted by the calcitonin receptor-like receptor (CLR), the receptor activity-modifying protein 1 (RAMP1) and a receptor component protein (RCP), all of which are necessary to form the functional CGRP receptor [12]. In the last decades, both preclinical and clinical evidence has accumulated supporting a role of CGRP in migraine mechanisms. First, CGRP levels are increased during a migraine attack [19] and in chronic migraine patients also in the pain-free interval [20], returning to normal levels after triptan administration associated with headache resolution [21,22,23,24]. Second, intravenous infusion of CGRP can induce migraine-like attacks in migraine patients [25,26]. Third, animal data suggest that CGRP can induce the generation of light intolerance, which may be considered a parallel of human photophobia, a phenomenon typically reported during migraine attack [27]. According to the abovementioned evidence, CGRP is considered a relevant target for migraine, and many efforts have been made to show that CGRP antagonism, by small molecules antagonists of CGRP receptor [28,29,30,31,32,33], an anti-CGRP receptor antibody [34,35,36] and anti-CGRP peptide antibodies [37,38,39,40,41,42,43,44,45] are all efficacious for the treatment or prevention of migraine.

## 4. Recent Findings on TRP Channels and Migraine Pain

While the mechanisms leading to the pain phase of migraine are unclear, the most likely explanation is that activation of nociceptors innervating the cranial meninges (and possibly the cerebral/meningeal vasculature) is responsible for the generation of headache [12,46]. Dilation of meningeal vessels on the headache side during migraine attacks triggered by cilostazol has recently been found, a finding that the authors suggest is an indicator of meningeal nociceptor activation [47], but this can only be taken as correlation and not causation of headache at this point. However, little information currently exists as to what specific mechanisms cause the activation of nociceptors in the meninges and/or near vessels. This includes a lack of knowledge of the extracellular events within the tissues themselves that occur during migraine attacks and the receptors expressed on nociceptors that respond to these events.

**Figure 1:**
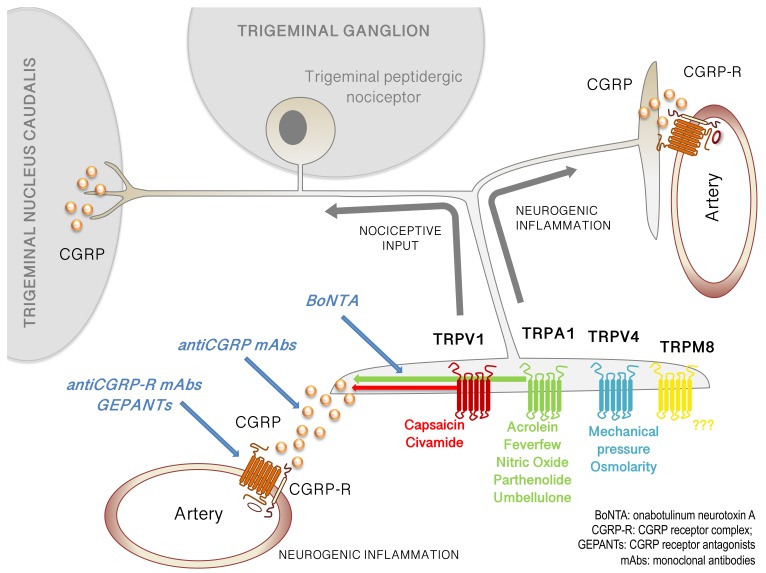
**TRP channels on trigeminal afferents and their potential role in migraine pathology.** Multiple TRP channels are expressed on trigeminal sensory neurons innervating the meninges including TRPV1, TRPA1, TRPV4, and TRPM8. These channels respond to stimuli implicated in migraine, both from a pathology perspective (e.g. acrolein on TRPA1) and a therapeutic perspective (e.g. parthenolide on TRPA1). Additional modulators are listed below their respective TRP channels. Activation of TRP channels on meningeal afferents leads to action potential signaling into the trigeminal nucleus caudalis (left) and ultimately to headache. Activation of TRP channels on these neurons also leads to the release of neuropeptides such as CGRP, activating CGRP receptors on blood vessels (right and bottom), causing vasodilation and contributing to neurogenic inflammation. Although not shown, TRP channels are also expressed on the central terminals of meningeal afferents, and CGRP is released as a transmitter in this synapse, both of which may also contribute to signaling within this circuit. Multiple migraine therapeutics may act in this circuit, including: BoNTA, which may indirectly contribute to decreased CGRP release and possibly inhibit recruitment of TRP channels to the membrane; GEPANTs, which block the CGRP receptor; antiCGRP mAbs, which sequester extracellular CGRP; and antiCGRP-R mAbs, which bind to and block the CGRP receptor. Abbreviations are found in the lower right corner.

Transient receptor potential (TRP) channels are a family of cation channels expressed primarily on the cell membrane that cluster into six families including TRPA, TRPC, TRPM, TRPP, TRPL, and TRPV [48,49,50]. These channels are likely to contribute to a number of different physiological processes ranging from thermosensation and pain to regulation of Ca^2+^ levels in the endoplasmic reticulum. Of relevance to the current discussion, they have been repeatedly hypothesized to contribute to migraine, specifically as an activation mechanism of meningeal nociceptors. Reasons for the interest in a TRP channel contribution to migraine are largely due to their expression on meningeal nociceptors and their responsiveness to a variety of endogenous and exogenous stimuli that may be of relevance to migraine attacks (Figure 1). Further, activation of TRP channels is well-known to promote the release of CGRP from sensory nerve endings, and they have been used extensively as probes for the function of CGRP in various processes [15,51,52]. Given the role of CGRP in migraine described above, and the as yet unclear mechanism by which CGRP is released during migraine, TRP channels remain a focus of interest for their potential contribution to attacks. A number of reviews on the topic of TRP channels and migraine have been published in recent years documenting the evidence that exists supporting their role in meningeal nociceptor activation [51,53,54,55,56,57,58], so only brief discussion of older work will be given here, while the focus will be on newer TRP channel migraine studies.

### 4.1. TRPV1

Based primarily on preclinical animal studies, it is clear that sensory neurons that innervate the meninges express TRPA1, TRPV1, TRPV4, and TRPM8 [51,53]. This is not likely to be an all-inclusive list as other TRP channels have not yet been extensively studied in this context. One of the first TRP channels to be investigated was TRPV1, which is activated by capsaicin and also sensitive to endocannabinoids, endovanilloids, lipoxygenase metabolites, nerve-growth factor, and prostaglandins, among other factors many of which may be relevant for migraine [48]. TRPV1 was hypothesized to be present on meningeal sensory fibers based on early studies with capsaicin application to the meninges (e.g. [59]). It was later directly demonstrated on these neurons using immunohistochemistry [60] and recently shown on nerve fibers in the arterial wall of scalp vessels from chronic migraine patients, where channel expression was higher compared to healthy controls [61]. Mechanisms leading to increased TRPV1 expression in chronic migraine patients are unclear, but preclinical studies using repeated 30-day administration of eletriptan or indomethacin found increased TRPV1 (and TRPA1) in the trigeminal ganglia, so migraine itself may upregulate the channel and/or migraine therapeutics may also have this potential [62].

There have been numerous studies using capsaicin and TRPV1 antagonists to probe meningeal afferent and vascular function and these studies suggest a role for the channel in headache mechanisms [51]. Recent studies using this approach have found differential capsaicin-induced meningeal blood flow changes in the presence of diet-induced obesity [63] in mice and suggest a role for TRPV1 signaling in the increased prevalence of migraine with obesity (consistent with the increase in capsaicin-induced nociception and photophobia with obesity [64]; a similar finding was also seen with TRPA1 [65]). Capsaicin stimulation of facial skin in human has also been used as a surrogate to study trigeminal-mediated dural vasodilation [66]. Using this assay, increased dermal blood flow was seen in female (but not male) migraine patients compared to controls, suggesting that changes occur in TRPV1 fiber neurovascular responses in migraine [67]; similar changes in migraine patients in capsaicin-induced dermal blood flow were also recently published [68].

The above studies still do not address the mechanisms by which TRPV1 may be activated in the meninges during migraine attacks, they only show that the function of the channel or TRPV1-expressing fibers are altered with migraine-like conditions. In one of the few recent studies addressing a potential mechanism for TRPV1 activation, the authors used two-photon in vivo imaging of anesthetized mouse to examine macrophage morphology and dendritic cell motility in the meninges in relation to cortical-spreading depression (CSD), which is thought to be the underlying basis of migraine aura and has been shown to activate meningeal nociceptors [69,70]. In time with CSD, meningeal macrophages changed to an active-like shape and migration of dendritic cells stopped [71]. The functional consequences of either of these events is not clear, but both cell types were found near TRPV1-expressing neurons, suggesting that CSD may alter meningeal immune cell function, which subsequently signal to this population of nociceptors. These studies are consistent with earlier findings on meningeal immune cells where CSD caused degranulation of mast cells [72], and together further implicate [73] changes in meningeal immune cell function during migraine as one mechanism of activation of dural nociceptors. Ultimately, while there may be a role for this channel in meningeal afferent signaling and in headache, the TRPV1 antagonist SB-705498 failed in a clinical migraine study [74]. This does not exclude a possibility for TRPV1 in migraine pathology, but it casts serious doubt on selective TRPV1 antagonism as a therapeutic approach and suggests that activation of TRPV1 alone is not sufficient to generate the headache phase of migraine.

### 4.2. TRPV4

The TRPV4 channel responds to a number of stimuli including changes in osmolarity (cell swelling) and the resulting mechanical forces imposed on the cell membrane [75,76,77], suggesting that this channel serves as part of a mechanosensory complex. It is thus interesting in the context of migraine as headache is known to be influenced by changes in intracranial pressure e.g. when coughing, sneezing, standing or sitting, or exercising [78]. TRPV4 is also expressed in the vasculature where its activity likely contributes to vascular permeability, among other functions [79]. Expression of TRPV4 on meningeal nociceptors was shown using application of channel activators to retrogradely-labeled trigeminal neurons in vitro, which produced currents consistent with TRPV4, and activation of this channel in the dura caused headache behavioral responses in rats [80]. Unfortunately, little work has been done directly examining TRPV4 and headache since this publication. TRPV4 function in sensory neurons is known to be modulated downstream of the protease-activated receptor 2 (PAR2) [81,82,83] and PAR2 activation sensitizes meningeal nociceptors to mechanical stimulation [84]. Until recently, preclinical behavioral studies had not been conducted with PAR2 activation in the meninges. Using more selective PAR2 agonists than those in prior studies, it was found that dural PAR2 activation causes headache behaviors in mice that were blocked by a selective PAR2 antagonist and absent in PAR2 knockout mice [85]. These studies provide further *indirect* evidence that TRPV4 activity on meningeal nociceptors may contribute to headache, but it remains unclear whether TRPV4 plays a direct role in the ability of meningeal afferents to detect pressure changes. More studies are needed to better explore the potential role for this channel in migraine.

### 4.3. TRPM8

Non-noxious cool temperature, natural products such as menthol, and synthetic compounds such as icilin are all known to activate TRPM8; this channel has been implicated in pain signaling in numerous prior studies [86,87]. It has also been the subject of much interest in the migraine community, initially based on the repeated identification of single-nucleotide polymorphisms (SNPs) and variants in the channel gene in numerous GWAS [53,87], most recently in a Taiwanese population study [88]. TRPM8 is found on trigeminal nerve fibers innervating the dura and appears to undergo developmental regulation where decreased expression occurs with age in mice such that relatively few neurons express TRPM8 in adult [89,90]. There have been conflicting reports as to what behavioral consequences are present when TRPM8 is activated in the meninges since studies have shown both increases [91] and decreases [89] in headache behavior in rodents following dural application of agonists. Ultimately, the context of when channel activation occurs may be relevant; if TRPM8 is activated alone it may cause headache as in the case of “ice cream headaches” while if it is activated along with ongoing inflammation, it may serve as an analgesic mechanism as is the case for topical menthol [53]. Consistent with this idea is a recent publication demonstrating that when TRPM8 is activated in the presence of an ongoing inflammatory state induced by a cocktail of inflammatory mediators, the effect was a reduction in headache-related behavior [92]. These authors also found increased overlap of TRPV1 and TRPM8 in the same trigeminal neurons innervating the dura in the presence of meningeal inflammation, suggesting changes in channel expression occur under these conditions. Interestingly, while TRPV1 and TRPM8 are not co-expressed in dorsal root ganglia [93], co-expression has been found in trigeminal neurons [94], although as mentioned above, its expression is subject to developmental regulation. Using a PC12 cell-based assay, they also found that TRPM8 activation could inhibit function of TRPV1 when both channels were expressed by the cells. Together, these new studies support the context-specific role of TRPM8 in migraine. The context of channel activation during native migraine attacks is not yet clear, so it remains unknown what role the channel plays. This latter point is particularly relevant for drug discovery as it is not known whether an agonist or antagonist would show efficacy for migraine. Nonetheless, Amgen recently published preclinical pharmacology and human safety data on a novel TRPM8 antagonist proposed to be tested for migraine [95].

One of the more intriguing studies related to TRPM8 and migraine in recent years was the finding that the TRPM8 SNP rs10166942 (either C or T at chr2:234835093), which has been identified in GWAS of migraine populations [53], is differentially expressed across the Earth depending on latitude [96]. It was previously reported that humans who carry the T;T allele have a higher risk of migraine than those carrying the rarer versions C;C or C;T [5,97]. The authors of this new study found that the frequency of T-allele expression was only 5% in populations native to Nigeria while it was 88% in those native to Finland, with South and East Asian populations between these extremes at 48% and 36%, respectively. Thus, T-allele frequency seems to be predicted by a latitudinal cline, suggesting that human migraine to the colder climates of Northern Europe introduced positive-selection pressure on TRPM8. Additionally, this further suggests that the variants in the channel may respond differentially to temperature (possibly with lower activity in colder climates which would facilitate adaptation to those environments), although it is not yet known what these variants do to channel function/expression. Nonetheless, the latitudinal cline of T-allele expression (higher in colder climates, lower in warmer climates) matches with the epidemiology of migraine which shows higher prevalence in Europe, lower prevalence in Africa, and intermediate prevalence in Asia (discussed in [96]). Better understanding of what the SNPs at rs10166942 do to the expression/function of TRPM8 will likely provide much insight into how the channel may be contributing to migraine, whether by greater or lesser activation during attacks.

### 4.4. TRPA1

The TRPA1 channel has probably received the most attention in preclinical headache/migraine studies as it is activated by an extensive list of endogenous and exogenous stimuli that may have relevance for migraine. These include reactive oxygen and nitrogen species, reactive prostaglandins, and many environmental irritants such as chlorine, formaldehyde, cigarette smoke, and acrolein [51,55,56,98]. For a recent review of TRPA1 and its role in numerous pain states, including migraine, see [58] in this same Special Issue of Pharmaceuticals. Although the TRPA1 activator mustard oil had been used as a probe to cause pain responses from the meninges in rodents for a number of years (see [99,100,101]), investigation of this channel in migraine expanded following a couple of key studies. One was the link between exposure to volatile oils from the *Umbellularia californica* tree (the “Headache Tree”) and headache attacks [102] which was followed by later studies showing that umbellulone activates TRPA1 and activation of TRPA1 in the dura by umbellulone and mustard oil causes headache responses in rats [103,104,105]. Umbellulone was also recently shown to facilitate propagation of CSD [106], so its actions, and that of TRPA1 in general, may not be restricted to peripheral sensory neurons and the meninges. This also led to several subsequent studies examining TRPA1-desensitizing compounds such as parthenolide from the feverfew herb and isopetasin from butterbur for their ability to desensitize meningeal nociceptors [107,108], potentially explaining the efficacy of these herbs in the treatment of migraine. Both of these studies show that these natural-product modulators of TRPA1 lead to functional desensitization of both the channel and the nerve ending expressing the channel. Consequences of this desensitization are decreased responses of nociceptive nerve endings to subsequent stimulation, which essentially acts as an inhibitor of neuronal function, and in the case of migraine, may lead to decreased noxious input from the meninges. The results of these studies suggest that compounds capable of desensitizing TRPA1 and TRPA1-expressing nerve endings may be viable new therapeutics for migraine.

The other key study leading to increased interest in TRPA1 and migraine was the finding that this channel mediates the vasodilatory response to inhalation of acrolein [109]. This was the first in a line of publications by these authors documenting and interaction between environmental irritants and TRPA1 that may lead to headaches following exposure. More recent work from these authors has shown that repeated exposure to acrolein potentiates meningeal vasodilatory responses to TRPA1 and TRPV1 activation [110] and leads to migraine-like cutaneous hypersensitivity and increased responses of the trigeminal afferent system to touch [111]. Further, lipidomic studies after acute or chronic inhalation of acrolein in rats found increased levels of a number of factors known to activate TRPV1 and TRPV4 [112]. These studies provide important mechanistic information for how repeated inhalation of environmental irritants can lead to headache disorders and also propose TRPA1 (as well as TRPV1 and TRPV4) antagonists as potential therapeutics in this area.

A number of studies over the last several years have been aimed at determining a role for TRPA1 in responses of the meningeal nociceptive system to other irritants such as hydrogen sulfide (H_2_S) as well as to nitric oxide (NO), both of which are well known to cause headaches. Importantly, these two compounds may act together as H_2_S and NO combine to form nitroxyl (HNO) that then reacts with and activates TRPA1 by covalent modification [113]. This reaction contributes to modulation of vascular tone downstream of H_2_S and NO via the HNO-activated and TRPA1-mediated release of CGRP. A recent study has demonstrated that this pathway contributes to activation of trigeminal afferent signaling [114] and can increase meningeal blood flow following topical administration if either NO or H_2_S donors onto the dura via the production of HNO [115]. However, this mechanism is far from fully explained as H_2_S-NO-TRPA1 pathway can also decrease activity in neurons of the spinal trigeminal nucleus, sometimes after prior activation [116]. Further, activation of TRPA1 alone (at least using acrolein) may not be sufficient to initiate afferent signaling from the meninges but may require co-activation of other channels such as TRPV1 [117]. Needless to say, these mechanisms are likely to be complex.

NO donors have long been recognized as migraine triggers, provoking attacks in approximately 75% of patients within 6 hours of administration [118,119]. However, the mechanisms by which this occurs remain poorly understood. Although one potential mechanism was described above (via formation of HNO), many other possibilities exist downstream of NO including activation of guanylyl cyclases, MAP kinases, nitrosylation of proteins, activation of cyclooxygenase enzymes, and production of peroxynitrite radicals [120,121,122,123,124,125]. As discussed above and also shown here [126], TRPA1 can mediate the nociceptive effects of NO, but given the diverse mechanisms downstream of NO and the diffusion of NO throughout all cells, its actions are likely complex. This question was extensively addressed recently using glyceryl trinitrate (GTN) administration to mice in the presence of pharmacological and genetic tools against TRPA1, aldehyde dehydrogenase, reactive oxygen and aldehyde species, and NADPH oxidase enzymes [127]. The authors examined both cutaneous facial vasodilation and allodynia following GTN and found that the initial vasodilation is TRPA1-independent, but is mediated by aldehyde dehydrogenase (which liberates NO from GTN) and a direct subsequent effect of NO on vessels. In contrast, the delayed allodynia following GTN was entirely dependent on TRPA1 expression. Activation of TRPA1 likely occurs due to direct NO-mediated interactions with the channel and also due to generation of reactive oxygen and carbonylic species from increased activity of NADPH oxidase in the trigeminal ganglion. Consistent with this study was another report showing that nitroglycerin can increase hyperalgesia in the orofacial formalin test in rats, an effect that was blocked by a TRPA1 antagonist, along with an increase in channel expression in the trigeminal ganglia, cervical spinal cord, and medulla [128]. These studies further implicates TRPA1 in migraine by directly demonstrating that it plays a role in headache-related behavior following exposure of one of the most reliable triggers of attacks.

## 5. BoNTA, CGRP and TRP Channels

Interestingly, recent evidence points to the possible existence of interconnections between the action of BoNTA, the release of CGRP and the TRP channels. In particular, in a rat model of inflammation induced by complete Freund’s adjuvant (CFA), it was demonstrated that pericranially-injected BoNTA is taken up by local sensory nerve endings, axonally transported to the trigeminal ganglion and transcytosed to dural afferents, where BoNTA-cleaved SNAP-25 colocalized with CGRP [129]. Importantly, BoNTA, similar to sumatriptan, reduced both the mechanical allodynia and the dural neurogenic inflammation evoked by CFA and this is likely to happen by the suppression of CGRP signalling [129]. Notably, it has been shown in migraine patients that CGRP levels after BoNTA treatment are significantly lower in comparison to the CGRP levels obtained before BoNTA treatment [130,131] and, more importantly, that the CGRP decrease is associated to the responsiveness to treatment. In fact, in responder patients, pretreatment CGRP levels were significantly higher than in nonresponders and, after treatment, the CGRP levels significantly decreased only in responders [130]. TRP channels, and especially TRPA1 and TRPV1, may also play a role in this scenario. It has been demonstrated that BoNTA inhibits TRPV1 trafficking to the plasma membrane in primary trigeminal ganglion neurons [132]. It has also been recently shown that the syntaxin 1-interacting protein, Munc18-1, is necessary for TRPV1-triggered CGRP release and for the response to the proinflammatory cytokine TNFα. Importantly, TNFα induces surface trafficking of TRPV1 and TRPA1 by means of a synaptic vesicle membrane protein, VAMP1, which is also essential for the exocytosis of CGRP. BoNTA inhibits the TNFα-elevated delivery and, consequently, abolishes the enhancement of Ca^2+^ influx through the upregulated surface-expressed TRPV1 and TRPA1 channels [133]. In addition, in a rodent model, pretreatment with BoNTA significantly reduced both capsaicin- and allyl isothiocyanate (AITC)-induced pain for at least 21 days, suggesting a durable pain-preventative effect of BoNTA due to decreased responsiveness of TRPV1 and TRPA1 toward their selective agonists [134]. Finally, BoNT-A administered to tissues outside the calvaria, is able to inhibit responses of C-type meningeal nociceptors to stimulation of their intracranial dural receptive fields with capsaicin or mustard oil. BoNTA effects on capsaicin responses are greater when the dose is injected along the suture lines, instead of being divided between muscles and sutures [135], which is interesting from a translational point of view given that BoNTA is injected in both locations in patients.

Although conclusive evidence is still needed, one may hypothesize that the abovementioned mechanisms related to TRP channels contribute to the decreased release of CGRP associated to BoNTA; initial evidence of the role of this circuitry has also been emerging for other drugs. Both valproic acid and topiramate have been shown to inhibit the capsaicin-induced elevation of CGRP immunoreactivity in the trigeminal ganglia and CGRP depletion in the dura mater of rats, even if with some differences among adult and pediatric rats [136]. Finally, the pretreatment with valproic acid has been shown to attenuate the enhanced blood flow responses observed after inhalation of the TRPA1 agonist acrolein in a rodent model of chronic migraine [111]. Together, these studies support the concept that decreases in TRP channel-mediated release of CGRP and other neuropeptides may contribute to the efficacy of many pharmacological agents for migraine.

## 6. Concluding Remarks

There is currently a great deal of excitement in the migraine field due to the approval of several, new CGRP-based therapeutics for use in humans. These novel agents add to the toolkit of the headache specialist and offer new options with distinct mechanisms of action for patients. However, it is already clear that these new therapeutics are not a panacea as many patients get suboptimal relief from the monoclonal antibodies (approximately 35% showing less than 50% relief in an open-label study of erenumab [137]; similar results have been observed in studies with other antibodies [138]). While mechanisms determining what patients will or will not experience relief with these CGRP therapies remain unclear, there is still great need for new therapeutics and TRP channels may represent additional targets for these new drugs. Questions remain regarding the viability of TRP channels as drug targets for migraine and these questions require additional work among the research community. Although TRPV1 antagonists failed in a prior human trial for migraine, there is still a clear role for TRPV1 in the activation of meningeal nociceptors and thus potential for alternate therapeutics to show efficacy. Intranasal administration of the TRPV1 agonist Civamide was efficacious in humans, likely via desensitization of nociceptors [139], providing proof of concept for TRPV1 modulating therapeutics, in this case with an agonist. Little work currently exists surrounding TRPV4 and migraine and this is the area that needs to most future exploration to evaluate this channel as a potential anti-migraine drug target. The repeated identification of TRPM8 variants in GWAS supports continued interest in this channel for the disorder, but it remains unknown whether therapeutics should be agonists or antagonists. Better understanding of the impact of the channel mutations on expression/function will help determine this answer. Finally, TRPA1 represents one of the most promising targets for new therapeutics based on its potential role in headache following exposure to endogenous substances, reactive oxygen species, environmental irritants, and migraine triggers such as NO donors as well as its potential role in treating attacks when desensitized by natural products. As of this writing, few TRPA1 antagonists have been developed for testing in humans, but there is now strong preclinical rationale for testing of these agents, although no clinical trials are currently conducted to this aim (www.clinicaltrials.gov accessed on February 17, 2019). Taken together, the data currently available surrounding TRP channels and migraine continue to make a compelling case for further development of new therapeutics based on modulation of these channels.

## Abbreviations

TRP	Transient-receptor potential
CGRP	Calcitonin gene-related peptide
BoNTA	onabotulinum toxin A
IHS	International Headache Society
ICHD	International Classification of Headache Disorders
GBD	Global Burden of Disease
NSAID	Non-steroidal anti-inflammatory drugs
ACE	Angiotensin-converting enzyme
GWAS	Genome-wide association study
SNP	Single nucleotide polymorphism
RAMP	Receptor-activity modifying protein
RCP	Receptor component protein
PAR-2	Protease-activated receptor type 2
H_2_S	Hydrogen sulfide
NO	Nitric oxide
HNO	Nitroxyl
MAP	Mitogen-activated protein
GTN	Glyceryl trinitrate
NADPH	Nicotinamide adenine dinucleotide phosphate
CFA	Complete Freund’s adjuvant
SNAP-25	Synaptosomal-associated protein 25
Munc	Mammalian uncoordinated
VAMP	Vesicle-associated membrane protein
TNF	Tumor necrosis factor
